# A cotransformation system of the unicellular red alga *Cyanidioschyzon merolae* with blasticidin S deaminase and chloramphenicol acetyltransferase selectable markers

**DOI:** 10.1186/s12870-021-03365-z

**Published:** 2021-12-04

**Authors:** Takayuki Fujiwara, Shunsuke Hirooka, Shin-ya Miyagishima

**Affiliations:** 1 Department of Gene Function and Phenomics, National Institute of Genetics, Mishima, Shizuoka, 411-8540 Japan; 2 Department of Genetics, Graduate University for Advanced Studies, SOKENDAI, Mishima, Shizuoka, 411-8540 Japan

**Keywords:** Cotransformation, *Cyanidioschyzon merolae*, Genetic modification, Photosynthetic eukaryote, Unicellular red alga

## Abstract

**Background:**

The unicellular red alga *Cyanidioschyzon merolae* exhibits a very simple cellular and genomic architecture. In addition, procedures for genetic modifications, such as gene targeting by homologous recombination and inducible/repressible gene expression, have been developed. However, only two markers for selecting transformants, uracil synthase (*URA*) and chloramphenicol acetyltransferase (*CAT*), are available in this alga. Therefore, manipulation of two or more different chromosomal loci in the same strain in *C. merolae* is limited.

**Results:**

This study developed a nuclear targeting and transformant selection system using an antibiotics blasticidin S (BS) and the BS deaminase (*BSD*) selectable marker by homologous recombination in *C. merolae*. In addition, this study has succeeded in simultaneously modifying two different chromosomal loci by a single-step cotransformation based on the combination of *BSD* and *CAT* selectable markers. A *C. merolae* strain that expresses mitochondrion-targeted *mSCARLET* (with the *BSD* marker) and *mVENUS* (with the *CAT* marker) from different chromosomal loci was generated with this procedure.

**Conclusions:**

The newly developed *BSD* selectable marker enables an additional genetic modification to the already generated *C. merolae* transformants based on the *URA* or *CAT* system. Furthermore, the cotransformation system facilitates multiple genetic modifications*.* These methods and the simple nature of the *C. merolae* cellular and genomic architecture will facilitate studies on several phenomena common to photosynthetic eukaryotes.

**Supplementary Information:**

The online version contains supplementary material available at 10.1186/s12870-021-03365-z.

## Background

The unicellular red alga *Cyanidioschyzon merolae* is an emerging model organism for studies on phenomena common to photosynthetic eukaryotes [1, 2]. The *C. merolae* nuclear and organellar genomes have been fully sequenced [3, 4]. The nuclear genome composition is very simple with very little genetic redundancy (16.5 Mbp; 4775 protein-coding genes). The cellular organization is also very simple; it contains a single nucleus, mitochondrion, chloroplast, and a minimal number of other membranous organelles [1, 2]. These organelles divide in a cell cycle-dependent manner, and cell cycle progression can be highly synchronized to a 12 h light/12 h dark cycle [1, 2, 5]. In addition, a genetic modification procedure via homologous recombination has been established. Based on this procedure, various genetic techniques, such as gene knockout [6], gene knock-in [7], stable expression of a transgene without any silencing activity [8], and inducible/repressible expression of an endogenous gene or transgene [9–11], have been developed. The combination of these genomic and cytological features and molecular genetic techniques in *C. merolae* has facilitated analyses on a variety of cellular phenomena in photosynthetic eukaryotes, such as cell cycle progression [7, 12], organelle division and inheritance [13–18], circadian rhythms [19], metabolism [20–22], photosynthetic apparatus [23–26], splicing [27], and epigenetics [28].

To manipulate the *C. merolae* nuclear genome, two kinds of selectable markers, uracil synthase (*URA*) gene [6, 8] and chloramphenicol (CP) acetyltransferase (*CAT*) gene [29, 30], have been developed. When multiple modifications on different chromosomal loci are required in a single strain, they can be achieved by a two-step transformation with *URA* and *CAT* markers [30] or by a marker recycling system, in which the *URA* marker is designed to be eliminated after the selection of transformants via intrachromosomal homologous recombination [31]. However, these sequential genetic modification methods are practically time-consuming (it takes ≥4 weeks for one round of genetic modification from transformation to obtain a liquid culture of a transformant before another round of genetic modification). To apply a *URA* selectable marker for genetic modification, the parental strain should be a uracil auxotrophic strain [32, 33]. Thus, a transformant generated using the *CAT* marker and wild-type (WT) cells as a parental strain can no longer be genetically modified. To overcome these limitations, this study developed a new drug-resistant selection system of *C. merolae* transformants that can work solely or in combination with the *CAT* marker system.

Blasticidin S (BS) was initially identified as an antibiotic inhibiting growth of the fungus *Piricularia oryzae*, a cause of rice plant disease [34]. The antibiotic is a nucleoside analogue (molecular wight 422) originally isolated from the bacterium *Streptomyces griseochromogenes* [34]. BS binds to the peptidyl transferase center of the large ribosomal subunit (50S and 60S in bacteria and eukaryotes, respectively) and thereby inhibits protein synthesis in eukaryotes and prokaryotes by interfering with elongation and termination step [35, 36].

BS deaminase (BSD) detoxifies BS by catalyzing the deamination of a cytosine moiety in BS [37–39]. BSD belongs to the deaminase superfamily that are involved in nucleotide and ADP-ribose metabolism, and distributed through in eukaryotes, bacteria, and phases [37]. The *BSD* gene was found only in the phyla Firmicutes and Actinobacteria in bacteria and fungi in eukaryotes [37], and cloned from the bacterium *Bacillus cereus* [40] and the fungi *Aspergillus terreus* [41] and has been used as a marker for selecting genetically manipulated strains in a variety of organisms, such as mammals [42], plants [40], yeasts [43], and the green alga *C. reinhardtii* [44], which do not possess *BSD* gene.

This study developed a transformant selection system using BS and *BSD* of *A. terreus* in *C. merolae*. In addition, the *BSD* selectable marker can be used in combination with the already developed *CAT* marker system to simultaneously modify two different chromosomal loci by a single-step cotransformation. These procedures will facilitate studies using multiple genetic modifications in *C. merolae*.

## Results and discussion

### Nuclear transformation system with the *BSD* selectable marker in *C. merolae*

**Fig. 1:**
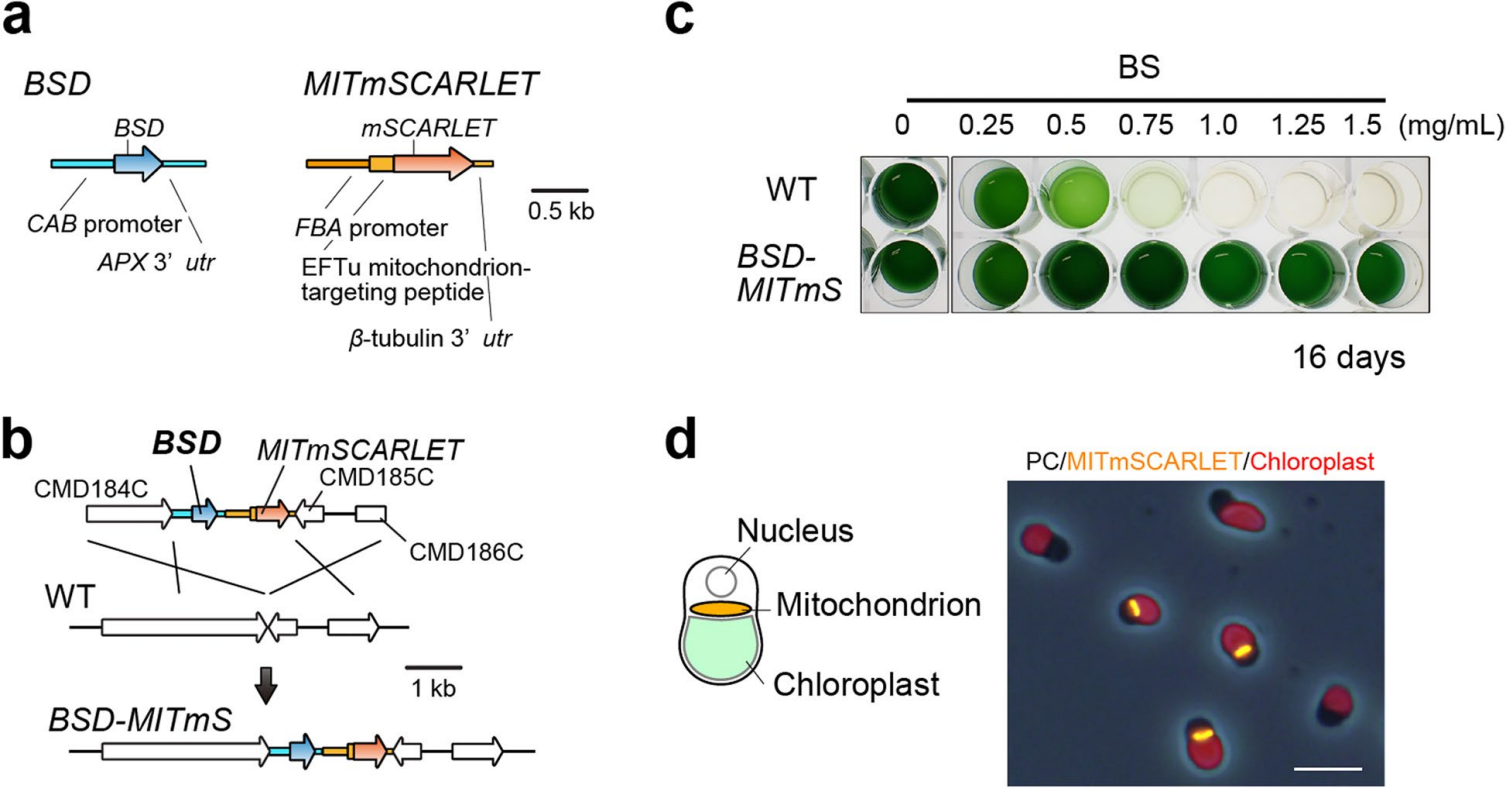
*C. merolae* transformation system using BS and *BSD* selectable marker. (**a**) Schematic diagram of the *BSD* selectable marker and *MITmSCARLET* expression cassette. To constitutively express *BSD* as a selectable marker in *C. merolae*, *BSD orf* of *A. terreus* was codon-optimized to the *C. merolae* nuclear genome and conjugated with the *C. merolae CAB* promoter and *APX* 3′-*utr*. To constitutively express *MITmSCARLET* (mSCARLET connected to the mitochondrion-targeted sequence of *C. merolae* EFTu) as a transgene, *MITmSCARLET orf* was conjugated with the *C. merolae FBA* promoter and β-tubulin 3′-*utr*. (**b**) Schematic diagram of the insertion of the *BSD* selectable marker and *MITmSCARLET* expression cassette into an intergenic region between CMD184C and CMD185C by homologous recombination. The first line indicates the introduced linear DNA, the second line indicates the genomic structure of the WT strain, and the third line indicates the expected genomic structure of the transformant (*BSD-MITmS*). (**c**) Selection of BS-resistant transformants in a liquid medium. After PEG-mediated transformation, cells were recovered in a drug-free MA2 liquid medium for 2 days in the light. Cells were transferred to the MA2 liquid medium supplemented with a series of BS concentrations and incubated for 16 days in the light in a 24-well plate. WT cells were also cultured as a negative control. (**d**) Fluorescent micrographs showing mSCARLET fluorescence detected in BS-resistant transformants in the medium supplemented with 1 mg/mL BS. Cells were observed 14 days after inoculation of cells into the medium. A schematic diagram of a *C. merolae* cell is also shown. The cell has a single disk-shaped mitochondrion and a single cup-shaped chloroplast. PC, phase-contrast; orange, fluorescence of mSCARLET; red, autofluorescence of the chloroplast. Bar, 5 μm

To validate the application of BS and *BSD* to the nuclear transformation of *C. merolae*, a selectable marker consisting of the promoter of *C. merolae CAB* gene (chlorophyll a/b binding protein; CMN234C), *BSD orf* of *A. terreus* (UniProtKB/Swiss-Prot ID of the amino acid sequence, P0C2P0.1; the nucleotide sequence was codon-optimized to the *C. merolae* nuclear genome), and 3′-*utr* of the *C. merolae APX* gene (ascorbate peroxidase; CMM158C) was constructed (Fig. 1a). In addition, an expression cassette of a transgene consisting of the promoter of the *C. merolae FBA* gene (fructose-1,6-biphosphate aldolase; CMM158C), sequence encoding mitochondrion-targeted peptide [N-terminal 77 amino acids [45]; of *C. merolae* EFTu (elongation factor thermo unstable; CMS502C)], *mSCARLET* (a monomeric red fluorescent protein) *orf*, and 3′-*utr* of the *C. merolae β*-tubulin gene (CMN263C) was constructed to fluorescently label the mitochondrion of transformant cells (Fig. 1a). The *CAB* and *FBA* promoters were chosen because a previous RNA-seq analysis showed that the mRNA abundance of these genes was relatively high in logarithmically growing *C. merolae* cells in the light [10]. The *BSD* selectable marker and the mitochondrion-targeted *mSCARLET* (*MITmSCARLET*) expression cassette were combined and sandwiched between *C. merolae* chromosomal sequences around CMD184C and CMD185C loci. This was done to integrate the *BSD* selectable marker and *MITmSCARLET* expression cassette into a chromosomal neutral locus between CMD184C and CMD185C [8] by homologous recombination (Fig. 1b). The resultant *C. merolae* transformant was named *BSD-MITmS*.

After introducing DNA to *C. merolae* WT cells by the polyethylene glycol (PEG)-mediated method [2, 46], cells were recovered for 2 days in an inorganic MA2 liquid medium without any drug in the light and transferred to the liquid medium with BS. To determine an appropriate BS concentration, WT cells and the *BSD-MITmS* transformant were cultured with a series of BS concentrations (0, 0.25, 0.5, 0.75, 1.0, 1.25, and 1.5 mg/mL) in the light for 16 days (Fig. 1c). As a result, WT cells died with BS in a dose-dependent manner, whereas the transformant grew in MA2 liquid medium supplemented with 1.0, 1.25, and 1.5 mg/mL BS (Fig. 1c). Fluorescence microscopy showed that mSCARLET fluorescence in the mitochondrion in most algal cells in the medium with 1.0 mg/mL BS, indicating that the transgene (*MITmSCARLET*) was successfully transformed into cells (Fig. 1d). However, some cells in the medium with 1.0 mg/mL BS lacked mSCARLET, suggesting that an unexpected integration of the *BSD* marker, such as truncation of the *MITmSCARLET* expression cassette, likely occurred in these nonfluorescent cells. Thus, we then tried to isolate clones with an expected integration of the selectable marker and the transgene.

### Selection and isolation of BS-resistant transformant clones on a gellan gum-solidified medium

**Fig. 2:**
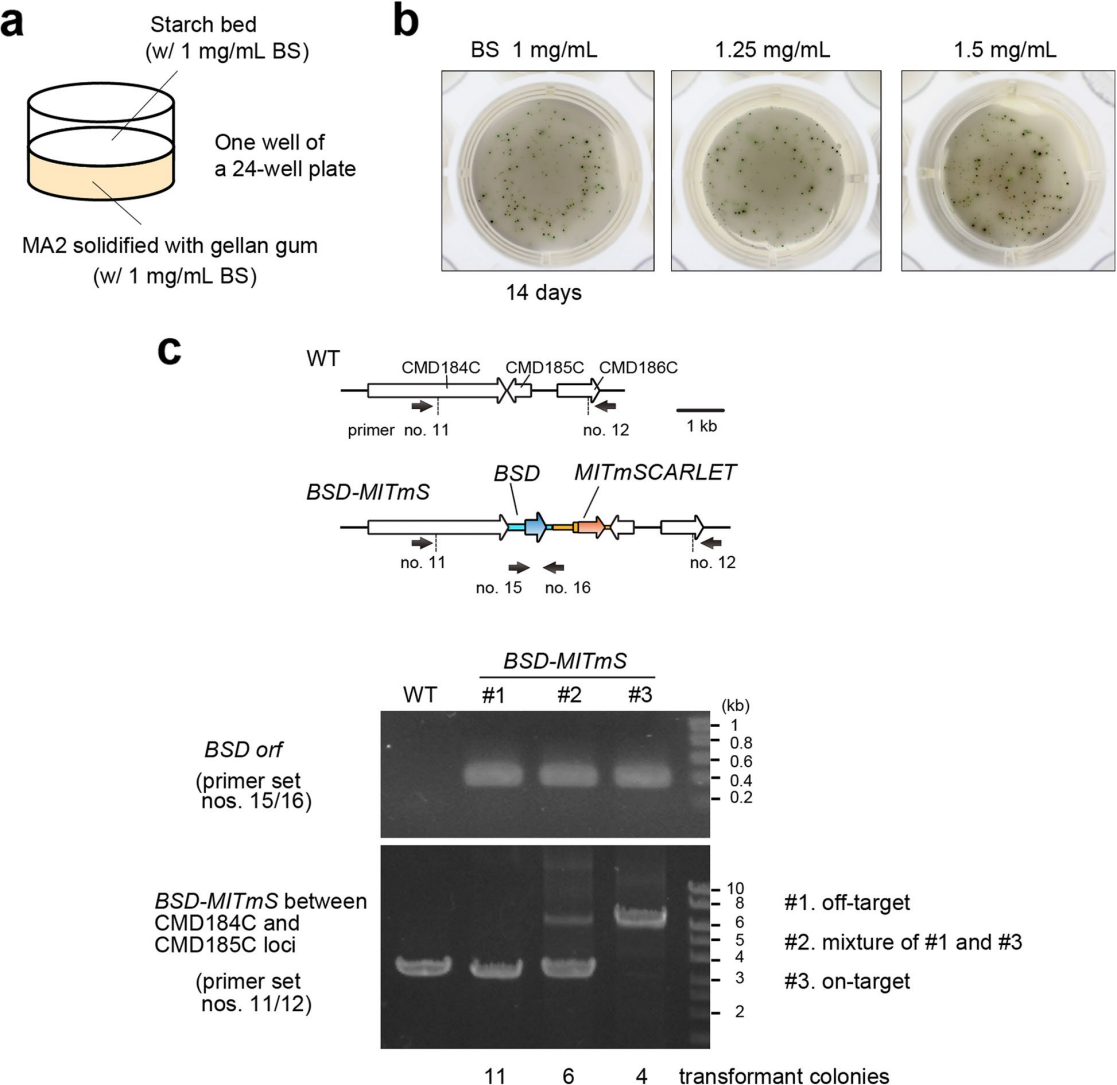
Selection and isolation of BS-resistant transformant clones on a gellan gum-solidified medium supplemented with BS. (**a**) Schematic diagram of the MA2 gellan gum plate supplemented with 1 mg/mL BS. MA2 gellan gum supplemented with 1 mg/mL BS was solidified in one well of a 24-well plate. The medium was covered with a cornstarch bed to facilitate the colony formation of *C. merolae*. (**b**) Colonies of BS-resistant clones on MA2 gellan gum plate supplemented with 1.0, 1.25 and 1.5 mg/mL BS. After PEG-mediated transformation, cells were recovered in a drug-free MA2 liquid medium for 2 days in the light. Cells were spread on MA2 gellan gum plate supplemented with BS and incubated for 14 days in the light. (**c**) Colony PCR analyses of BS-resistant clones. The positions of the primers are indicated as arrows below the chromosomal structure of the WT and *BSD-MITmS* transformant. For detection of the *BSD* marker insertion either by off-target and on-target insertion (the primer set, nos. 15/16), the predicted size of the PCR product was 0.4 kb*.* In the PCR amplifying CMD184C-CMD186C loci (the primer set, nos. 11/12), the predicted size of the PCR product of on-target insertion of the *BSD-MITmS* construct was 6.3 kb and the size for off-target insertion or the WT chromosome was 3.3 kb. The exact positions and sequences of the primers are indicated in Supplementary Table S1. Full length unprocessed gel image of Fig. 2c is shown in Supplementary Fig. S1a.

A gellan gum-solidified MA2 medium supplemented with BS was prepared. The medium was covered with a cornstarch bed (Fig. 2a) to facilitate the colony formation of *C. merolae* [6, 8]. After the PEG-mediated introduction of DNA into cells, cells were recovered in MA2 liquid medium in the light for 2 days. Then, 50 μL of the culture, which was diluted to give a concentration of OD_750_ = 0.01, were inoculated onto the cornstarch bed with BS and incubated in a CO_2_ (3%) incubator at 42 °C in the light. After incubation for 14 days, colonies appeared on the BS gellan gum plate (Fig. 2b).

To verify the targeted insertion of the *BSD* marker and *MITmSCARLET* into the intergenic region between CMD184C and CMD185C, polymerase chain reaction (PCR) analyses of the colonies were performed. Typical examples of electrophoretic patterns of the PCR products are shown in Fig. 2c and Supplementary Fig. S1a. The PCR analyses showed that the *BSD* marker was integrated into the genome of all transformant colonies examined (21/21 colonies; #1-#3 on the upper gel in Fig. 2c and Supplementary Fig. 1a). In contrast, the PCR analysis amplifying the CMD184C-CMD186C loci showed three patterns depending on the transformant colony; a single band showing off-target insertion of the transgene (11/21 colonies; #1 on the lower gel in Fig. 2c and Supplementary Fig. S1a), two bands showing on-target and off-target insertions of the transgene (6/21 colonies; #2 on the lower gel in Fig. 2c and Supplementary Fig. S1a), and a single band showing on-target insertion of the transgene (4/21 colonies; #3 on the lower gel in Fig. 2c and Supplementary Fig. S1a).

### Simultaneous modification of two different chromosomal loci by a single-step cotransformation with *BSD* and *CAT* selectable markers in *C. merolae*

**Fig. 3:**
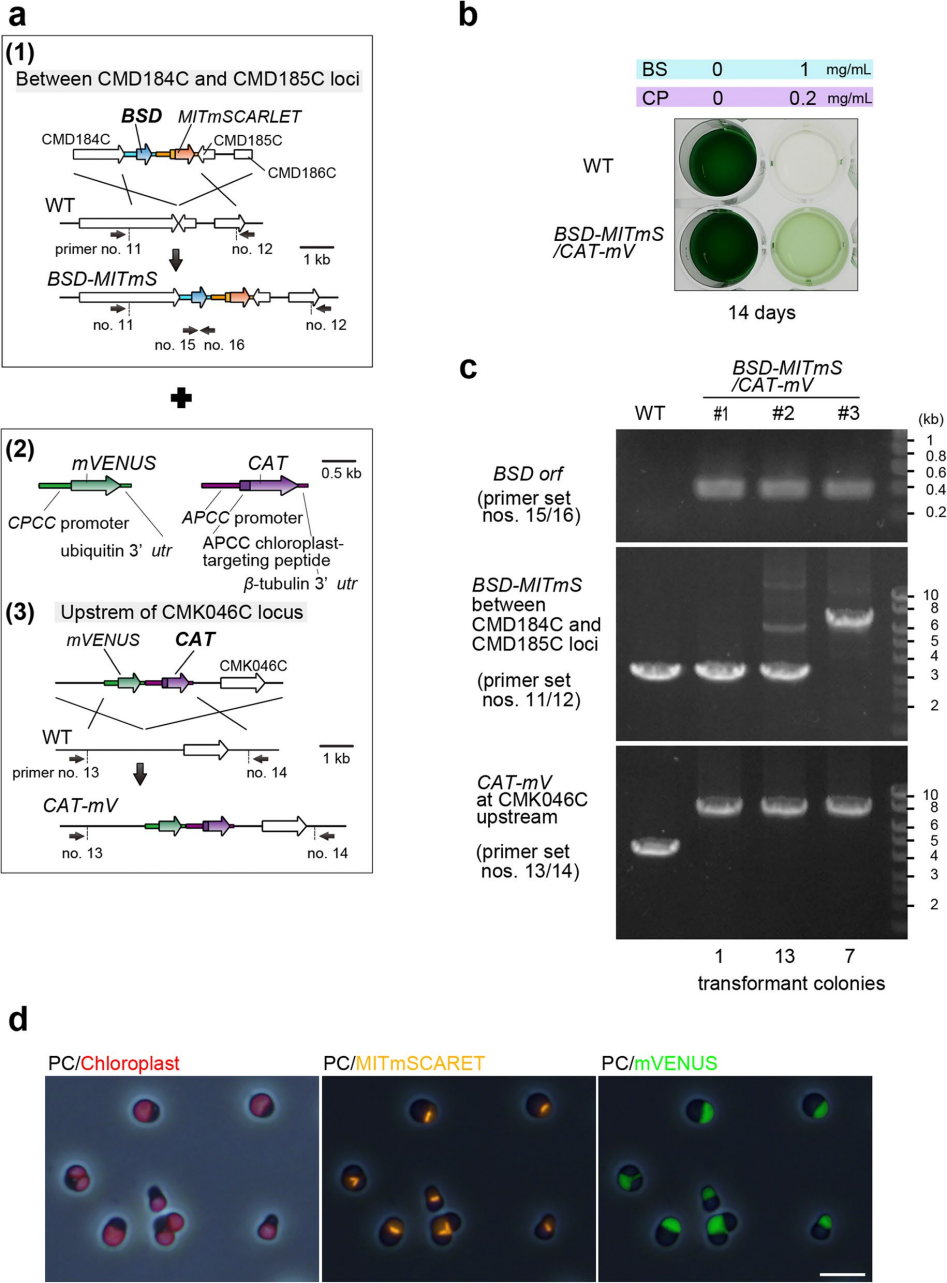
Simultaneous modification of two different chromosomal loci by a single-step cotransformation with *BSD* and *CAT* selectable markers in *C. merolae.* (**a**) Schematic diagram of the insertion of the *mVENUS* transgene with the *CAT* selectable marker and that of the *MITmSCARLET* transgene with the *BSD* selectable marker into two different chromosomal loci by homologous recombination. (1) Schematic diagram of the insertion of the *MITmSCARLET* transgene with the *BSD* selectable marker as in Fig. 1a. (2) Schematic diagram of the *mVENUS* expression cassette and the *CAT* selectable marker. To constitutively express *mVENUS*, *mVENUS orf* was conjugated with the *C. merolae CPCC* promoter and ubiquitin 3′-*utr*. To constitutively express the *CAT* selectable marker, *CAT orf* was conjugated with the *C. merolae APCC* promoter and β-tubulin 3′-*utr*. (3) Schematic diagram of the insertion of the *mVENUS* transgene with the *CAT* selectable marker into a chromosomal neutral locus. The *mVENUS* transgene was connected with the *CAT* selectable marker and integrated into the chromosomal region at CMK046C upstream. The first line indicates the introduced linear DNA, the second line indicates the genomic structure of the WT chromosome, and the third line indicates the expected genomic structure of the transformant (*CAT-mV*). (**b**) Selection of BS- and CP-resistant transformants in MA2 liquid medium supplemented with BS and CP. After PEG-mediated transformation of the two constructs, cells were recovered in a drug-free MA2 liquid medium for 2 days in the light. Cells were transferred to MA2 liquid medium supplemented with 1 mg/mL BS and 0.2 mg/mL CP and incubated for 14 days in the light in a 24-well plate. WT cells were also cultured as a negative control. (**c**) Colony PCR analyses of BS- and CP-resistant clones. The primer positions are indicated in (**a)**. The transformants in the liquid culture above were spread onto a drug-free MA2 gellan gum plate to generate colonies of transformant clones before the PCR analyses. For detection of the *BSD* marker insertion either by off-target and on-target insertion (the primer set, nos. 15/16), the predicted size of the PCR product was 0.4 kb*.* In the PCR amplifying CMD184C-CMD186C loci (the primer set, nos. 11/12), the predicted size of the PCR product of on-target insertion of the *BSD-MITmS* construct was 6.3 kb and the size for off-target insertion or the WT chromosome was 3.3 kb. In the PCR amplifying the CMK046C locus and its upstream (the primer set, nos. 13/14), the predicted size of the PCR product of on-target insertion of the *CAT-mV* construct was 7.5 kb and the size for off-target insertion or the WT chromosome was 4.3 kb. The exact positions and sequences of the primers are indicated in Supplementary Table S1. Full length unprocessed gel images of Fig. 3c are shown in Supplementary Fig. S1b. (**d**) Fluorescent micrographs showing mSCARLET fluorescence in the mitochondrion (MITmSCARLET, orange) and mVENUS (green) fluorescence in the cytosol in the *BSD-MITmS/CAT-mV* transformant clone. PC, phase-contrast; red, autofluorescence of the chloroplast. Bar, 5 μm

To develop an efficient and multiple genetic modification system in *C. merolae*, two different chromosomal loci were modified simultaneously using both *BSD* and *CAT* selectable markers. In addition to *BSD*-*MITmS* (Figs. 1b and 3a), another construct consisting of the *CAT* selectable marker and *mVENUS* transgene (*CAT-mV*) was prepared and designed to be integrated into an upstream region of CMK046C locus as another chromosomal neutral locus [10] (Fig. 3a). *BSD*-*MITmS* and *CAT-mV* were simultaneously introduced to *C. merolae* WT cells by the PEG-mediated method (Fig. 3a). After recovery in drug-free MA2 liquid medium for 2 days, transformants were selected in MA2 liquid medium supplemented with both BS (1 mg/mL) and CP (0.2 mg/mL) rather than gellan gum-solidified medium. This was because, in a previous study, CP-resistant transformants could grow in MA2 liquid medium supplemented with CP but were not able to form colonies on MA2 gellan gum plate supplemented with CP [30]. After incubation in MA2 liquid medium supplemented with BS and CP in the light for 14 days, WT died, whereas the transformant survived (Fig. 3b). The transformant culture was spread and incubated on a drug-free MA2 gellan gum plate to generate single colonies. PCR analyses of the colonies showed that the *BSD* marker was integrated into the genome of all transformant colonies examined (21/21 colonies; #1-#3 on the upper gel in Fig. 3c and Supplementary Fig. S1b). *BSD*-*MITmSCARLET* was inserted into the intergenic region between CMD184C and CMD185C by on-target insertion in some colonies (7/21 colonies; #3 on the middle gel in Fig. 3c and Supplementary Fig. S1b) and the *CAT- mV* vector was inserted into the intergenic region of CMK046C upstream by on-target insertion in all colonies examined (21/21 colonies; #1, #2, and #3 on the lower gel in Fig. 3c and Supplementary Fig. S1b). In the transformant clones, in which both transgenes were inserted by on-target insertion (#3), fluorescence microscopy showed that the mitochondrion was labeled with mSCARLET fluorescence, and the cytosol emitted mVENUS fluorescence (Fig. 3d). These results demonstrated that cotransformation is feasible in *C. merolae*.

In the preset study, the efficiency of on-target insertion of the *BSD-MITmS* construct was lower than that of the *CAT-mV* construct. Because the *BSD* marker was inserted into the genome by off-target insertion in some transformant clones, further optimization of the homology arms in the *BSD-MITmSCARLET* construct would be required to improve the targeting efficiency.

In the cotransformation system described above, it took 2 weeks to select BS and CP-resistant cells in a liquid medium after transformation and another 2 weeks to obtain single colonies of intended transformants on a gellan gum plate, which was faster than the previous procedures for a two-step sequential transformation [30, 31]. In addition, the newly developed *BSD* selectable marker will enable an additional genetic modification to the already generated *C. merolae* transformants based on the *URA* or *CAT* system. Besides these advantages, a large-sized DNA will likely be introduced into a chromosome by conjugating *BSD* and *CAT* markers to the respective borders of DNA. Multiple genetic modifications will also be useful for metabolic engineering because such modifications often require the inactivation of two or more enzymatic genes [47].

## Conclusions

This study has developed a transformant selection method using BS and *BSD* in *C. merolae*. In addition, a cotransformation system has been developed to simultaneously modify two different chromosomal loci by a combination of *BSD* and *CAT* selectable markers. These methods and the simple nature of *C. merolae* cellular and genomic architecture will facilitate studies on several phenomena in photosynthetic eukaryotes.

## Methods

### Algal culture

*C. merolae* 10D WT (NIES-3377), *BSD-MITmS*, and *BSD-MITmS/CAT-mV* strains were maintained in MA2 liquid medium [2] [(NH_4_)_2_SO_4_ 40 mM, KH_2_PO_4_ 8 mM, MgSO_4_ 4 mM, CaCl_2_ 1 mM, FeCl_3_ 100 μM, EDTA-2Na 72 μM, ZnCl_2_ 2.8 μM, MnCl_2_ 16 μM, Na_2_MoO_4_ 7.2 μM, CuCl_2_ 1.3 μM, and CoCl_2_ 0.7 μM; the pH was adjusted to 2.3 with H_2_SO_4_] in 60 mL tissue culture flasks (TPP Techno Plastic Products AG), with agitation at 120 rpm under continuous white light (30 μmol·m^− 2^·s^− 1^) at 42 °C.

### Preparation of linear DNA for *C. merolae* transformation

The sequences and primers used in this study are listed in Supplementary Table S1. Linear DNA for the transformation of *C. merolae* was prepared as described below.

To generate the *C. merolae BSD*-*MITmS* strain, DNA that contained the *BSD* selectable marker and *MITmSCARLET* expression cassette was prepared. For the homologous recombination at an intergenic region between CMD184C and CMD185C loci in the nuclear genome, the *BSD* selectable marker and *MITmSCARLET* expression cassette were tandemly connected and sandwiched between sequences identical to CMD184C and CMDl85C loci, respectively (Fig. 1b), as described below. DNA fragment I [a portion of CMD184C *orf* (773rd nucleotide to the stop codon) flanked with the 25 bp downstream sequence] was amplified by PCR with the primer set #1/2 using *C. merolae* WT genomic DNA as a template. DNA fragment II consisting of the *CAB* (CMN234C) promoter, *BSD orf*, and *APX* (CMM158C) 3′-*utr* and DNA fragment III consisting of the *FBA* (CMI049C) promoter, sequence encoding the mitochondrion-targeting peptide of EFTu (CMS502C), *mVENUS orf* (codon-optimized to *C. merolae* nuclear genome), and β-tubulin (CMN263C) 3′-*utr* were commercially synthesized. DNA fragment IV (sequence from the 28th to 1880th nucleotide downstream of CMD184C *orf*; this sequence corresponded to CMD185 and a portion of CMD186) was amplified by PCR with the primer set #3/4 using *C. merolae* WT genomic DNA as a template. DNA fragments I to IV were assembled and cloned into the plasmid pUC19 vector (TAKARA, Japan) using the In-Fusion HD cloning kit (TAKARA) for amplification in *Escherichia coli*. The assembled DNA fragments I to IV were amplified by PCR with the primer set #5/6 to prepare linear DNA for *C. merolae* transformation. The PCR product was purified using the QIAquick PCR Purification Kit (Qiagen).

To generate the *C. merolae BSD*-*MITmS/CAT-mV*, DNA containing the *CAT* selectable marker and *mVENUS* expression cassette was prepared*.* For homologous recombination at an intergenic region at CMK046C upstream, the tandemly connected *CAT* selectable marker and *mVENUS* expression cassette were sandwiched between two genomic sequences around the CMK046C locus as described below. DNA fragment V (a chromosomal sequence from 2300 to 898 bp upstream of CMK046C *orf*) was amplified by PCR with the primer set #7/8 using *C. merolae* WT genomic DNA as a template. DNA fragment VI consisting of *CPCC* (CMP166C) promoter, *mVENUS orf*, and ubiquitin (CMK296C) 3′-*utr* and DNA fragment VII consisting of *APCC* (CMO250C) promoter, sequence encoding the chloroplast-targeting peptide of APCC, *CAT orf*, and *β*-tubulin 3′-*utr* were commercially synthesized. DNA fragment VIII (CMK046C *orf* flanked with 897 bp upstream and 471 bp downstream sequences) was amplified by PCR with the primer set #9/10 using *C. merolae* WT genomic DNA as a template. DNA fragments V to VIII were assembled and cloned into the plasmid pUC19 vector using the In-Fusion HD cloning kit for amplification in *E. coli*. The assembled DNA fragments V to VIII were amplified by PCR with the primer set #5/6 to prepare linear DNA for *C. merolae* transformation. The PCR product was purified using the QIAquick PCR Purification Kit.

### Transformation of *C. merolae*

The transformation of *C. merolae* was carried out by the PEG-mediated method [46], as described previously [2, 30], with minor modifications. To generate the *BSD*-*MITmS* strain, 4 μg of *BSD*-*MITmS* PCR product was introduced into *C. merolae* WT. To generate the *BSD*-*MITmS/CAT-mV* strain, 4 μg each of *BSD*-*MITmS* and *CAT-mV* PCR products were mixed (8 μg in total) and introduced into *C. merolae* WT.

The transformed cells were cultured for 2 days in 8 mL of MA2 liquid medium in one well of a 6-well plate (VIOLAMO) in a CO_2_ (3%) incubator at 42 °C in the light (40 μE) for recovery and selected on an MA2 gellan gum plate supplemented with 1, 1.25, and 1.5 mg/mL BS (the preparation procedure is described below) or 2 mL MA2 liquid medium supplemented with both 1 mg/mL BS and 0.2 mg/mL CP (the concentration was defined previously [30];) in one well of a 24-well plate (TPP Techno Plastic Products). Colony PCR analysis was carried out to verify targeted insertions of constructs into the intergenic regions between CMD184C and CMD185C loci and upstream of the CMK046C locus using the primer sets #11/12 and #13/14, respectively. The *BSD* marker integration into the genome in transformants was verified by PCR using the primer set #15/16.

### MA2 gellan gum plate supplemented with BS

The MA2 gellan gum plate was prepared, as described previously [8], with minor modifications. One milliliter of 1%  gellan gum (FUJIFILM, Japan) solution (autoclaved to dissolve gellan gum in water) and 1 mL of 2× MA2 liquid medium were poured into one well of a 24-well plate. Immediately, a stock solution of BS (Cayman Chemical, USA; 50 mg/mL in distilled water) was added to the mixture at a final concentration of 1.0, 1.25, and 1.5 mg/mL and mixed. After gellan gum had solidified, 0.5 mL of 40% slurry of cornstarch, suspended in MA2 liquid medium with 1, 1.25, and 1.5 mg/mL BS, was placed on the solidified medium (Fig. 2a). After cornstarch had sedimented onto the gellan gum plate, excess suspension solution was removed, and cornstarch was dried up on a clean bench.

### Microscopy

Images of cells were captured using a fluorescence microscope (BX51; Olympus) equipped with a three-charge-coupled device camera system (DP71; Olympus). To detect mVENUS and mSCARLET fluorescence, narrow-band filter sets (U-MNIBA3; Olympus and XF37; Omega, respectively) were used.

## Supplementary Information

**Additional file 1: **Supplementary **Figure S1**. Full length unprocessed gel images of Figs. 2c and 3c.

**Additional file 2: **Supplementary **Table S1**. List of nucleotide sequences of primers and synthetic DNA used in this study.

## Data Availability

The plasmids and *C. merolae* transformant strains generated in the current study are available from the corresponding authors upon request. The nucleotide sequences of *C. merolae* genetic loci (ID: CMN234C/*CAB*, CMM158C/*APX*, CMI049C/*FBA*, CMS502C/*EFTu*, CMN263C/β-tubulin, CMP166C/*CPCC,* CMO250C/*APCC*, CMD184C, CMD185C, CMD186C and CMK046C) used during the current study are available in *Cyanidioschyzon merolae* Genome Project v3 (http://czon.jp/).

## Abbreviations

BS: Blasticidin S
BSD: Blasticidin S deaminase
CAT: Chloramphenicol acetyltransferase
CP: Chloramphenicol
PEG: Polyethylene glycol

## References

[1] Miyagishima SY, Tanaka K. The unicellular red alga *Cyanidioschyzon merolae*—the simplest model of a photosynthetic eukaryote. Plant Cell Physiol. 2020(0):1–16. doi:10.1093/pcp/pcab052. 10.1093/pcp/pcab052 PMC8504449 33836072

[2] Kuroiwa T, Miyagishima S, Matsunaga S, Sato N, Nozaki H, Tanaka K, Misumi M. *Cyanidioschyzon merolae*. A new model eukaryote for cell and organelle biology.. Springer Nature Singapore Pte Ltd. 2017:17–41. doi:10.1007/978-981-10-6101-1

[3] Matsuzaki M, Misumi O, Shin-I T, Maruyama S, Takahara M, Miyagishima SY, Mori T, Nishida K, Yagisawa F, Nishida K (2004). Genome sequence of the ultrasmall unicellular red alga *Cyanidioschyzon merolae* 10D. Nature..

[4] Nozaki H, Takano H, Misumi O, Terasawa K, Matsuzaki M, Maruyama S, Nishida K, Yagisawa F, Yoshida Y, Fujiwara T (2007). A 100%-complete sequence reveals unusually simple genomic features in the hot-spring red alga *Cyanidioschyzon merolae*. BMC Biol.

[5] Suzuki K, Ehara T, Osafune T, Kuroiwa H, Kawano S, Kuroiwa T (1994). Behavior of mitochondria, chloroplasts and their nuclei during the mitotic cycle in the ultramicroalga *Cyanidioschyzon merolae*. Eur J Cell Biol.

[6] Imamura S, Terashita M, Ohnuma M, Maruyama S, Minoda A (2010). Nitrate assimilatory genes and their transcriptional regulation in a unicellular red alga *Cyanidioschyzon merolae*: genetic evidence for nitrite reduction by a sulfite reductase-like enzyme. Plant Cell Physiol..

[7] Fujiwara T, Tanaka K, Kuroiwa T, Hirano T (2013). Spatiotemporal dynamics of condensins I and II: evolutionary insights from the primitive red alga *Cyanidioschyzon merolae*. Mol Biol Cell.

[8] Fujiwara T, Ohnuma M, Yoshida M, Kuroiwa T, Hirano T (2013). Gene targeting in the red alga *Cyanidioschyzon merolae*: single- and multi-copy insertion using authentic and chimeric selection markers. PLoS One.

[9] Sumiya N, Fujiwara T, Kobayashi Y, Misumi O, Miyagishima SY (2014). Development of a heat-shock inducible gene expression system in the red alga *Cyanidioschyzon merolae*. PLoS One.

[10] Fujiwara T, Kanesaki Y, Hirooka S, Era A, Sumiya N, Yoshikawa H, Tanaka K, Miyagishima SY (2015). A nitrogen source-dependent inducible and repressible gene expression system in the red alga *Cyanidioschyzon merolae*. Front Plant Sci.

[11] Fujiwara T, Hirooka S, Ohbayashi R, Onuma R, Miyagishima SY (2020). Relationship between cell cycle and diel transcriptomic changes in metabolism in a unicellular red alga. Plant Physiol.

[12] Kobayashi Y, Kanesaki Y, Tanaka A, Kuroiwa H, Kuroiwa T, Tanaka K (2009). Tetrapyrrole signal as a cell-cycle coordinator from organelle to nuclear DNA replication in plant cells. Proc. Natl. Acad. Sci. U S A..

[13] Miyagishima SY, Nishida K, Mori T, Matsuzaki M, Higashiyama T, Kuroiwa H, Kuroiwa T (2003). A plant-specific dynamin-related protein forms a ring at the chloroplast division site. Plant Cell.

[14] Nishida K, Takahara M, Miyagishima SY, Kuroiwa H, Matsuzaki M, Kuroiwa T (2003). Dynamic recruitment of dynamin for final mitochondrial severance in a primitive red alga. Proc. Natl. Acad. Sci. U S A..

[15] Yoshida Y, Kuroiwa H, Misumi O, Yoshida M, Ohnuma M, Fujiwara T, Yagisawa F, Hirooka S, Imoto Y, Matsushita K, Kawano S, Kuroiwa T (2010). Chloroplasts divide by contraction of a bundle of nanofilaments consisting of polyglucan. Science..

[16] Fujiwara T, Kuroiwa H, Yagisawa F, Ohnuma M, Yoshida Y, Yoshida M, Nishida K, Misumi O, Watanabe S, Tanaka K, Kuroiwa T (2010). The coiled-coil protein VIG1 is essential for tethering vacuoles to mitochondria during vacuole inheritance of *Cyanidioschyzon merolae*. Plant Cell.

[17] Yagisawa F, Fujiwara T, Ohnuma M, Kuroiwa H, Nishida K, Imoto Y, Yoshida Y, Kuroiwa T (2013). Golgi inheritance in the primitive red alga, *Cyanidioschyzon merolae*. Protoplasma.

[18] Imoto Y, Abe Y, Honsho M, Okumoto K, Ohnuma M, Kuroiwa H, Kuroiwa T, Fujiki Y (2018). Onsite GTP fuelling via DYNAMO1 drives division of mitochondria and peroxisomes. Nat Commun.

[19] Miyagishima SY, Fujiwara T, Sumiya N, Hirooka S, Nakano A, Kabeya Y, Nakamura M (2014). Translation-independent circadian control of the cell cycle in a unicellular photosynthetic eukaryote. Nat Commun.

[20] Imamura S, Kanesaki Y, Ohnuma M, Inouye T, Sekine Y, Fujiwara T, Kuroiwa T, Tanaka K (2009). R2R3-type MYB transcription factor, CmMYB1, is a central nitrogen assimilation regulator in *Cyanidioschyzon merolae*. Proc. Natl. Acad. Sci. U S A..

[21] Moriyama T, Sakurai K, Sekine K, Sato N (2014). Subcellular distribution of central carbohydrate metabolism pathways in the red alga *Cyanidioschyzon merolae*. Planta..

[22] Takahashi S, Okubo R, Kanesaki Y, Zhou B, Takaya K, Watanabe S, Tanaka K, Imamura S (2021). Identification of transcription factors and the regulatory genes involved in triacylglycerol accumulation in the unicellular red alga *Cyanidioschyzon merolae*. Plants (Basel).

[23] Krupnik T, Kotabová E, van Bezouwen LS, Mazur R, Garstka M, Nixon PJ, Barber J, Kaňa R, Boekema EJ, Kargul J (2013). A reaction center-dependent photoprotection mechanism in a highly robust photosystem II from an extremophilic red alga, *Cyanidioschyzon merolae*. J Biol Chem.

[24] Antoshvili M, Caspy I, Hippler M, Nelson N (2019). Structure and function of photosystem I in *Cyanidioschyzon merolae*. Photosynth Res.

[25] Pi X, Tian L, Dai HE, Qin X, Cheng L, Kuang T, Sui SF, Shen JR (2018). Unique organization of photosystem I-light-harvesting supercomplex revealed by cryo-EM from a red alga. Proc. Natl. Acad. Sci U S A.

[26] Haniewicz P, Abram M, Nosek L, Kirkpatrick J, El-Mohsnawy E, Olmos JDJ, Kouřil R, Kargul JM (2018). Molecular mechanisms of Photoadaptation of photosystem I Supercomplex from an evolutionary Cyanobacterial/algal intermediate. Plant Physiol.

[27] Stark MR, Dunn EA, Dunn WS, Grisdale CJ, Daniele AR, Halstead MR, Fast NM, Rader SD (2015). Dramatically reduced spliceosome in *Cyanidioschyzon merolae*. Proc. Natl. Acad. Sci U S A..

[28] Mikulski P, Komarynets O, Fachinelli F, Weber APM, Schubert D (2017). Characterization of the Polycomb-group mark H3K27me3 in unicellular algae. Front Plant Sci.

[29] Zienkiewicz M, Krupnik T, Drożak A, Golke A, Romanowska E (2017). Chloramphenicol acetyltransferase-a new selectable marker in stable nuclear transformation of the red alga *Cyanidioschyzon merolae*. Protoplasma..

[30] Fujiwara T, Ohnuma M, Kuroiwa T, Ohbayashi R, Hirooka S, Miyagishima SY (2017). Development of a double nuclear gene-targeting method by two-step transformation based on a newly established chloramphenicol-selection system in the red alga *Cyanidioschyzon merolae*. Front Plant Sci.

[31] Takemura T, Imamura S, Kobayashi Y, Tanaka K (2018). Construction of a selectable marker recycling system and the use in epitope tagging of multiple nuclear genes in the unicellular red alga *Cyanidioschyzon merolae*. Plant Cell Physiol..

[32] Minoda A, Sakagami R, Yagisawa F, Kuroiwa T, Tanaka K (2004). Improvement of culture conditions and evidence for nuclear transformation by homologous recombination in a red alga, *Cyanidioschyzon merolae* 10D. Plant Cell Physiol.

[33] Taki K, Sone T, Kobayashi Y, Watanabe S, Imamura S, Tanaka K. Construction of a *URA5.3* deletion strain of the unicellular red alga *Cyanidioschyzon merolae*: A backgroundless host strain for transformation experiments J. Gen. Appl. Microbiol. 2015;61(5):211–214. doi: 10.2323/jgam.61.211. 10.2323/jgam.61.211 26582291

[34] Takeuchi S, Hirayama F, Uera F, Sakai H, Yonehara H. Blasticidin S, a new antibiotic. J Antibiot (Tokyo) 1958;11(1):1–5. 13525246

[35] Svidritskiy E, Ling C, Ermolenko DN, Korostelev A. A Blasticidin S inhibits translation by trapping deformed tRNA on the ribosome Proc Natl Acad Sci USA 1993;110(30):12283–12288. doi: 10.1073/pnas.1304922110. 10.1073/pnas.1304922110 PMC3725078 23824292

[36] Powers KT, Stevenson-Jones F, Yadav SKN, Amthor B, Bufton JC, Borucu U, Shen D, Becker JP, Lavysh D, Hentze MW, Kulozik AE, Neu-Yilik G, Schaffitzel C (2021). Blasticidin S inhibits mammalian translation and enhances production of protein encoded by nonsense mRNA. Nucleic Acids Res.

[37] Iyer LM, Zhang D, Rogozin IB, Aravind L (2011). Evolution of the deaminase fold and multiple origins of eukaryotic editing and mutagenic nucleic acid deaminases from bacterial toxin systems. Nucleic Acids Res.

[38] Seto H, Ôtake N, Yonehara H (1966). Biological transformation of Blasticidin S by *Aspergillus fumigatus* sp. Agric Biol Chem.

[39] Endo T, Furuta K, Kaneko A, Katsuki T, Kobayashi K, Azuma A, Watanabe A, Shimazu A. Inactivation of blasticidin S by *Bacillus cereus*. I. inactivation mechanism. J. Antibiot. (Tokyo). 1987;40(12):1791–1793. doi: 10.7164/antibiotics.40.1791 10.7164/antibiotics.40.1791 3123450

[40] Kamakura T, Yoneyama K, Yamaguchi I (1990). Expression of the blasticidin S deaminase gene (bsr) in tobacco: fungicide tolerance and a new selective marker for transgenic plants. Mol Gen Genet.

[41] Kimura M, Kamakura T, Tao QZ, Kaneko I, Yamaguchi I (1994). Cloning of the blasticidin S deaminase gene (BSD) from Aspergillus terreus and its use as a selectable marker for *Schizosaccharomyces pombe* and *Pyricularia oryzae*. Mol Gen Genet.

[42] Izumi M, Miyazawa H, Kamakura T, Yamaguchi I, Endo T, Hanaoka F (1991). Blasticidin S-resistance gene (bsr): a novel selectable marker for mammalian cells. Exp Cell Res.

[43] Fukuda H, Kizaki Y (1999). A new transformation system of *Saccharomyces cerevisiae* with blasticidin S deaminase gene. Biotechnol Lett.

[44] Félix de Carpentier, Jeanne Le Peillet, Nicolas D. Boisset, Pierre Crozet, Stéphane D. Lemaire, and Antoine Danon. Blasticidin S Deaminase: A New Efficient Selectable Marker for *Chlamydomonas reinhardtii*. Front Plant Sci. 2020;11:242. doi: 10.3389/fpls.2020.00242 10.3389/fpls.2020.00242 PMC7066984 32211000

[45] Imoto Y, Kuroiwa H, Yoshida Y, Ohnuma M, Fujiwara T, Yoshida M, Nishida K, Yagisawa F, Hirooka S, Miyagishima SY, Misumi O, Kawano S, Kuroiwa T (2013). Single-membrane-bounded peroxisome division revealed by isolation of dynamin-based machinery. Proc. Natl. Acad. Sci. U S A..

[46] Ohnuma M, Yokoyama T, Inouye T, Sekine Y, Tanaka K (2008). Polyethylene glycol (PEG)-mediated transient gene expression in a red alga, *Cyanidioschyzon merolae* 10D. Plant Cell Physiol..

[47] Alper H, Jin YS, Moxley JF, Stephanopoulos G (2005). Identifying gene targets for the metabolic engineering of lycopene biosynthesis in *Escherichia coli*. Metab Eng.

