# Supplementary figures and images for: A cotransformation system of the unicellular red alga Cyanidioschyzon merolae with blasticidin S deaminase and chloramphenicol acetyltransferase selectable markers
Source: BMC Plant Biol. 2021 Dec 4;21:573. doi: 10.1186/s12870-021-03365-z (PMC8642924; doi:10.1186/s12870-021-03365-z)

Supplementary Figure S1

a Original gel image of Figure 2c

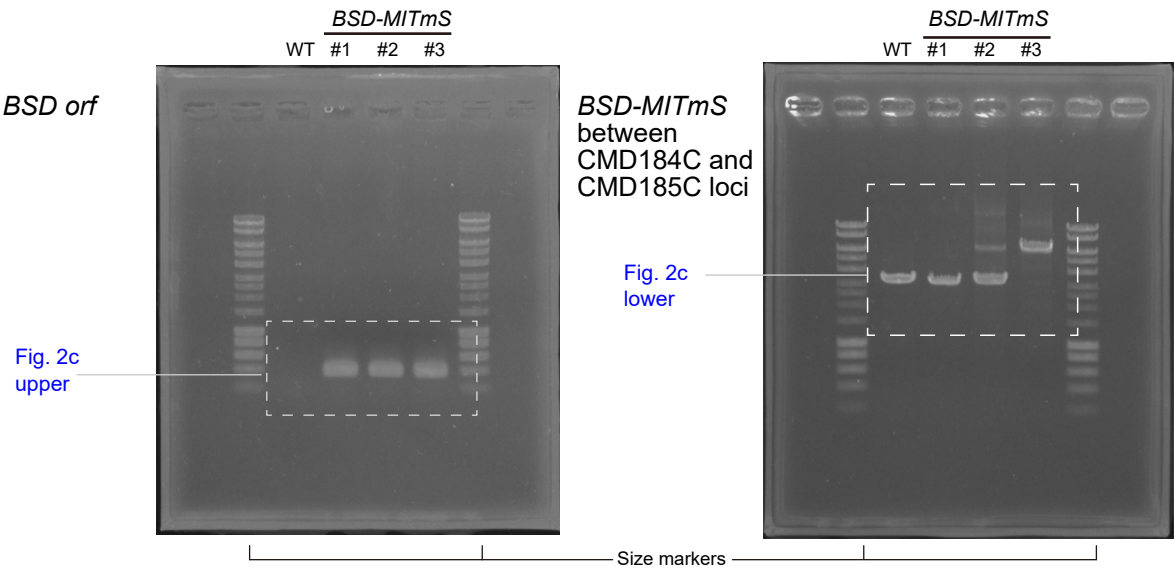

b Original gel images of Figure 3c

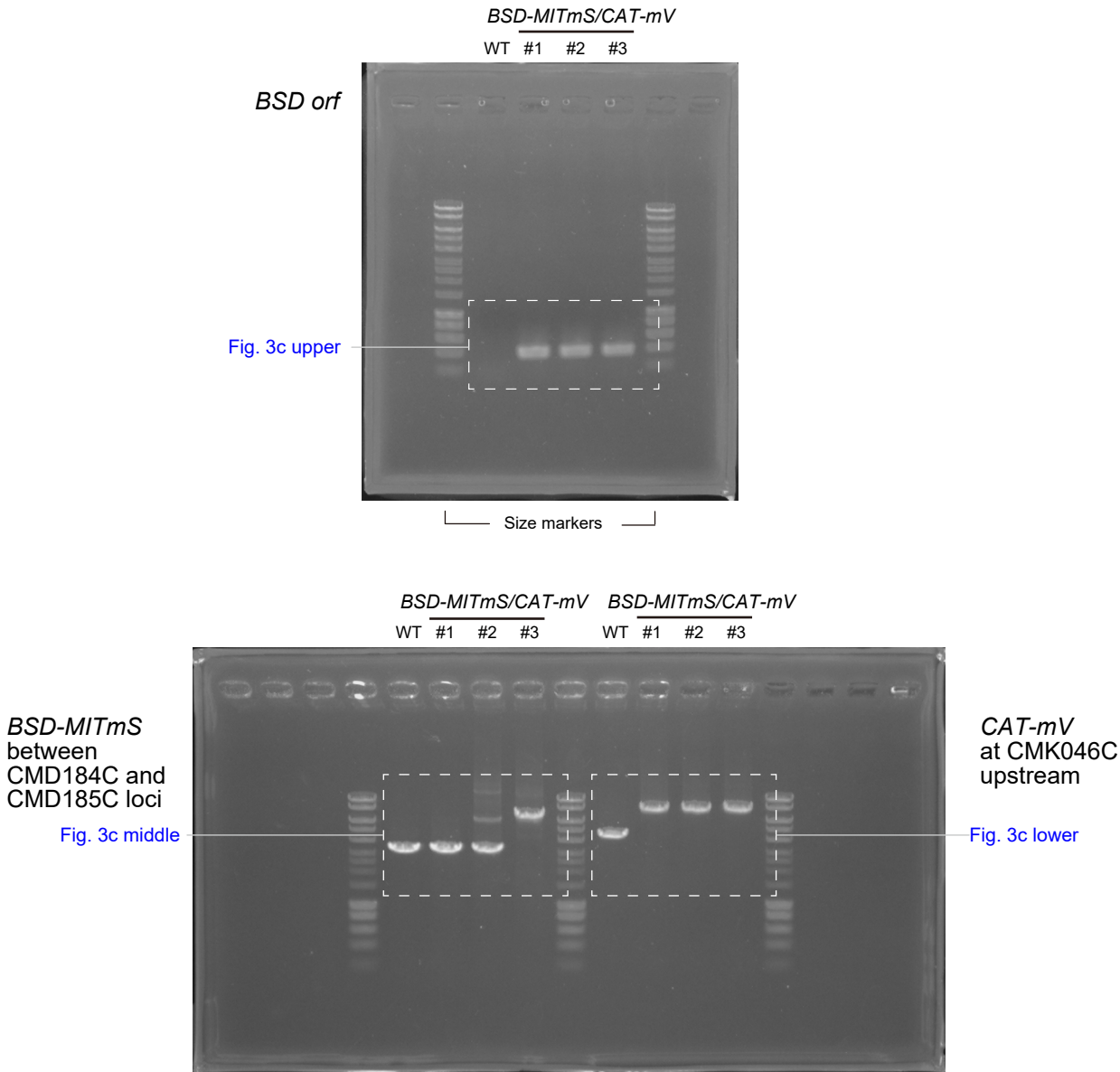

Supplement: Supplementary file 1 — Additional file 1: Supplementary Figure S1. Full length unprocessed gel images of Figs. 2c and 3c. [file 12870_2021_3365_MOESM1_ESM.pdf]
